# Distinguishing Different Varieties of Oolong Tea by Fluorescence Hyperspectral Technology Combined with Chemometrics

**DOI:** 10.3390/foods11152344

**Published:** 2022-08-05

**Authors:** Yan Hu, Youli Wu, Jie Sun, Jinping Geng, Rongsheng Fan, Zhiliang Kang

**Affiliations:** College of Mechanical and Electrical Engineering, Sichuan Agriculture University, Ya’an 625000, China

**Keywords:** oolong tea, classification, spectroscopy, chemometrics

## Abstract

Oolong tea is a semi-fermented tea that is popular among people. This study aims to establish a classification method for oolong tea based on fluorescence hyperspectral technology(FHSI) combined with chemometrics. First, the spectral data of Tieguanyin, Benshan, Maoxie and Huangjingui were obtained. Then, standard normal variation (SNV) and multiple scatter correction (MSC) were used for preprocessing. Principal component analysis (PCA) was used for data visualization, and with tolerance ellipses that were drawn according to Hotelling, outliers in the spectra were removed. Variable importance for the projection (VIP) > 1 in partial least squares discriminant analysis (PLS–DA) was used for feature selection. Finally, the processed spectral data was entered into the support vector machine (SVM) and PLS–DA. MSC_VIP_PLS–DA was the best model for the classification of oolong tea. The results showed that the use of FHSI could accurately distinguish these four types of oolong tea and was able to identify the key wavelengths affecting the tea classification, which were 650.11, 660.29, 665.39, 675.6, 701.17, 706.31, 742.34 and 747.5 nm. In these wavelengths, different kinds of tea have significant differences (*p* < 0.05). This study could provide a non-destructive and rapid method for future tea identification.

## 1. Introduction

Tea is one of the most popular nonalcoholic beverages in the world and is widely loved by people [1]. Oolong tea belongs to semi-fermented tea, which is one of the most popular beverages in China [2]. According to a report on China’s tea consumption market in 2021, the output of five of six traditional Chinese tea categories, except yellow tea, increased by varying degrees. The output of oolong tea was 287,200 tons, with an increase of 9400 tons over last year [3]. Oolong tea has certain health functions, such as anti-ageing, anti-atherosclerosis and the prevention of diabetes, and it even affects weight loss [4]. There are many types and brands of oolong tea, and the price of different kinds of tea varies greatly. Tieguanyin is the best oolong tea and the most expensive oolong tea on the market. Therefore, illegal businessmen often use ordinary oolong tea (similar in appearance to Tieguanyin) to counterfeit Tieguanyin for sale and deceive consumers, which seriously endangers the order of the tea market [5]. Ordinary oolong teas include Benshan, Maoxie and Huangjingui, which are similar in appearance to Tieguanyin and often appear in the market as an alternative tea to Tieguanyin [6]. The price of Tieguanyin is usually tens or even hundreds of times higher than that of ordinary tea [7]. Therefore, it is very necessary to classify oolong tea [5].

The traditional identification of tea varieties usually relies on human sensory evaluation. However, sensory evaluation has some obvious drawbacks [8]: Relying heavily on human subjective consciousness, the judging process is time-consuming and may damage the tea samples. For large batches of samples, this method is not conducive to large-scale testing, and even professional judging experts can only test a limited number of samples per day [9]. With the development of testing technology, chemical analysis methods are becoming increasingly common [10], such as gas chromatography–mass spectrometry (GC–MS) [11,12], inductively coupled plasma mass spectrometry (ICP-MS), and atmospheric solids analysis probe–mass spectrometry (ASAP–MS) [6]. Tan et al. [6] reported that they used ASAP–MS to authenticate Chinese oolong tea. The results showed that it was possible to classify oolong tea using ASAP–MS and PCA–K-nearest neighbor (KNN) models with high accuracy of up to 100%. Wang et al. [13] reported an aroma-based method for distinguishing different grades of Nongxiang Tieguanyin. Unknown samples can be classified by comparing the spatial distribution of unknown samples with known standards in PCA or hierarchical cluster analysis (HCA). Even if these chemical analysis methods are accurate, this work of identifying teas is still time-consuming and complex and requires professionals to complete. In addition to chemical analysis methods, the use of sensors is also a good way to identify tea varieties [14]. The electronic tongue and electronic nose [15] mimic mammalian species identification through taste and smell, and this approach yields qualitative information about the sample by responding nonspecifically to the chemical of interest and analyzing its response through an appropriate pattern recognition procedure [16]. Chen et al. [17] reported an improved classification of oolong tea with different varieties by combining two novel artificial sensing tools (i.e., gustatory sensors and olfactory sensors). The results show that the discrimination capability of the combined system is superior to that obtained with the two sensors systems separately, and eventually, linear discriminant analysis (LDA) achieved a 100% classification rate by cross-validation. This method achieves high accuracy; however, the validation of this method is subject to many uncertainties during the tea testing, with the possibility of temperature and humidity changes and aroma volatilization, which requires a high experimental environment. In addition, this method may damage the structure of tea samples. Because of the shortcomings of the above methods, it is urgent to develop a rapid and nondestructive detection method [18].

As a nondestructive testing method, spectroscopy has been widely used in tea species identification and quality testing [19,20]. Firmani et al. [21] coupled NIR spectroscopy with PLS–DA and soft independent modelling of class analogies (SIMCA). Both provided satisfactory results in discriminating PGI samples from the other teas and adulterated Darjeeling. Ren et al. [22] reported that a visible-near-infrared (Vis-NIR) spectrometer and support vector machine (SVM)-based kernels were used for the qualitative categorization of black tea. It demonstrated that Vis-NIR spectroscopy can be a rapid, inexpensive, efficient, alternative method for predicting the quality of black tea.

Fluorescence hyperspectral imaging (FHSI) technology breaks through the traditional analytical methods to obtain images and fluorescence spectral information of samples [23], providing a new idea for nondestructive and rapid detection [24]. FHSI has been applied to mineral identification [25], apple quality detection [26], rice origin identification [27] and contamination monitoring and classification of cotton [28]. Therefore, the combination of FHSI and tea classification has great research potential. This technique is used to obtain a fluorescent hyperspectral image of tea by shining incident light at a specific wavelength, which causes the absorption of light from the ground state to the excited state and immediately excites the emitted light [3]. The method has a short detection time and does not damage the sample itself, making it a good detection tool for species differentiation due to the specificity of the sample spectra [3].

In this study, the fluorescence hyperspectral images of four oolong teas (a total of 216 tea samples) have been acquired. The extracted spectral data have been preprocessed by MSC and SNV and visualized by PCA, and the outliers have been screened out by drawing tolerance ellipses according to Hotelling. Then, the feature wavelengths with VIP > 1 in PLS-DA have been selected, and finally, the processed spectra have been input into two discriminant models, SVM and PLS–DA, for the prediction of classification results. In addition, this study also analyzes the key wavelengths that could affect the classification of oolong teas.

## 2. Materials and Methods

### 2.1. Tea Samples

Tieguanyin is usually difficult to distinguish from Huangjingui, Benshan and Maoxie in actual sales. These are some of the reasons we chose Tieguanyin and these three types of oolong teas. First, by appearance, all the selected teas have a dense particle appearance. Huangjingui is slightly more distinguishable than the other three teas in colour, with a more pronounced yellow colour. For Maoxie, some of it has white hairy clusters in appearance, but this feature does not appear in all teas. Benshan is a close relative of Tieguanyin, known as the brother of Tieguanyin, and is one of the four well-known oolong teas in China. Second, in terms of economic value, Tieguanyin sells for hundreds or even thousands of RMB per 500 g in the market, while other teas sell for no more than a hundred RMB per 500 g.

All samples for this experiment were obtained from Anxi County, Quanzhou City, Fujian Province, China. To ensure the accuracy of the samples, tea samples were purchased from trusted merchants, and professional tea appraisers were invited for identification. After the identification was completed, the tea samples were sent to the fluorescence hyperspectral laboratory for spectral data acquisition. A total of 216 oolong tea samples (Each type has 54 samples) were obtained. Each sample weighed 5 g and was packaged in individual bags, and all samples were kept in a cool and dry environment.

### 2.2. Data Acquisition

The GaiaFluo(/Pro)-VH-HR series fluorescence hyperspectral test system produced by Jiangsu Dualix Technology Co., Ltd.(Wuxi, China) The system consists of a dark box, a xenon light source, an excitation filter, an emission filter, a hyperspectral camera and supporting software. The hyperspectral camera has a spectral range of 400–1000 nm and a resolution of 2.8 nm. There are five excitation filters (357, 390, 452, 534 and 628 nm) and five fluorescence filters (475, 495, 530, 570 and 610 nm). It was found that the 390 nm excitation filter was better able to cut off the input of other wavelengths. After several times of fluorescence filter selection, it was found that the fluorescence intensity began to show a significant wave at 500 nm, so the 475 nm fluorescence filter was finally selected, and the separation of the fluorescence signal from other parasitic light could be better accomplished under this filter, thus capturing the best fluorescence image [29]. Then, spectral data of ROI were extracted through ENVI 5.3.

### 2.3. Spectral Preprocessing

**Figure 1:**
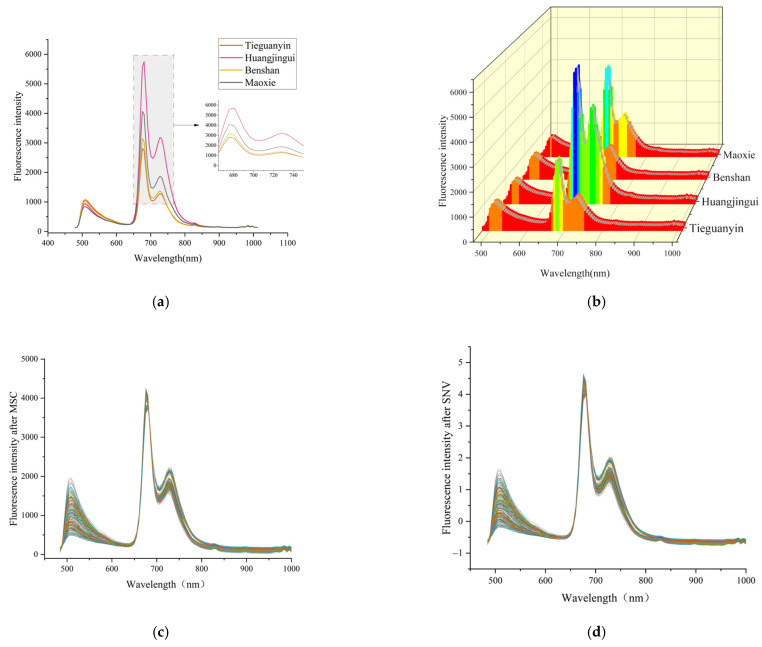
(**a**) Average spectra of four oolong teas; (**b**) three-dimensional plot of the spectral curves; (**c**) spectra after MSC; (**d**) spectra after SNV.

The fluorescence hyperspectral imaging system was used to obtain the spectral data of the four oolong teas, as shown in Figure 1a,b. Since the selected fluorescence filter was 475 nm, the figure shows that the data were filtered in the wavelength before 475 nm, and the spectral data between 475–1100 nm were finally retained. Before data modelling, it is crucial to preprocess the spectral data, which can effectively reduce noise and baseline drift in the spectra. In this study, the spectra were preprocessed using multiple scatter correction (MSC) and standard normal variation (SNV). MSC is performed by linearly fitting each spectrum to a reference spectrum, separating the additive and multiplicative effects of the measurement. It is an important step in correcting for scattered light based on different grain sizes. SNV is a mathematical transformation of log(1/R) spectra to remove slope variations and correct for scattering effects. Each spectrum is first centred on the spectral value, and then the centre spectrum is scaled according to the standard deviation calculated for each spectral value [30].

### 2.4. Principal Component Analysis

Principal component analysis (PCA) is a commonly used multivariate statistical method [13] that is performed by generating a set of principal components that are linear transformations of the original variables; these new principal components are orthogonal to each other and ranked according to the explained variance [31]. PCA scoring plots are often used for visualization and can provide a clear view of the sample distribution. In addition, in the PCA score plot, the tolerance ellipse is drawn according to Hotelling, and the observations far from the ellipse are outliers so that the distribution of the data in the lower dimension can be observed by PCA, and the outliers in the sample can be excluded from it [32].

### 2.5. VIP (Variable Importance for the Projection) in PLS–DA

With a total of 126 wavelengths in the spectral data, there may be a large amount of redundant information in high dimensions, which is not conducive to modelling. In order to exclude the interference of irrelevant variables, the key wavelengths that affect the classification effect are identified. PLS–DA, as a stable discriminant statistical method, is suitable for cases with a large number of explanatory variables, double covariance, a small number of sample observations and high interference noise. Additionally, VIP in PLS–DA is a method that can quantify the contribution of each variable to the classification [13]. Used to explain the significance of X and the variables associated with Y, the VIP for each wavelength position is calculated by weighting the sum of squares of the PLS loading weights with the sum of squares explained in each model component [8], and the sum of squares of all VIPs is equal to the model. The sum of squares of all VIPs is equal to the number of terms in the model. Hence, the average VIP is equal to 1. VIP values larger than 1 indicate “important” X-variables, and values lower than 0.5 indicate “unimportant” X-variables. The interval between 1 and 0.5 is a gray area, where the importance level depends on the size of the data set. [33]. The larger the VIP, the more significant the difference between the different kinds of oolong tea is. In this study, those with VIP > 1 were considered to be the important contributors to tea classification.

### 2.6. Classification Methods

This study is designed for the classification problem of oolong tea. Two classification methods, PLS–DA and SVM, are chosen for model building. PLS–DA [34], as a stable discriminant statistical method, is suitable for cases with a large number of explanatory variables, double covariance, a small number of sample observations and high interference noise. SVM is a classification method that dominates in solving small samples and nonlinear and high-dimensional data [23].

### 2.7. Evaluation Index

In this paper, the performance of the model is evaluated using accuracy, recall and precision sensitivity [35,36]. They are defined in Equations (1)–(3).

(1) $$Accuracy=\frac{TP+TN}{TP+TN+FP+FN}$$

(2) $$Recall=\frac{TP}{TP+FN}$$

(3) $$Precision=\frac{TN}{TN+FP}$$

where *TP* is true positive, *TN* is true negative, *FP* is false positive, *FN* is false negative.

Pretreatment, PCA and PLS–DA(VIP) were performed using SIMCA v14.1 (Soft Independent Modelling by Class Analogy, Umetrics, Umea, Sweden). All classification models were built-in Python 3.8. ANOVA was performed using SPSS v21.0 (SPSS Inc., Chicago, IL, USA).

## 3. Results and Discussions

### 3.1. Spectral Characteristics

Figure 1 shows the spectral curves of oolong tea, where 1(a) is the average spectra of the four teas. The figure demonstrates the relationships between fluorescence intensity and the spectral wavelengths of the four teas, indicating that all of them belong to the oolong tea family and have similar internal compositions and roughly the same overall spectral trends. There is a clear distinction between the spectral wavelengths of 500–850 nm, in which the fluorescence intensity of Tieguanyin is higher than the other three teas, which is influenced by catechins, theaflavins and anthocyanins [5]. In the interval of 650–850 nm, the fluorescence intensity of Tieguanyin is lower than the other three teas, while Huangjingui is a slightly yellow tea among the four kinds of tea, and the fluorescence intensity is twice as high as that of Tieguanyin. The spectra are bimodal in the 600–800 nm interval, appearing at 690 nm and 735 nm, respectively [3]. This is where the influence of the pigments in the tea is most likely, mainly as a result of the combined effects of chlorophyll and carotenoids [3]. Figure 1b is a three-dimensional plot of the spectral curve, in which the distribution between the spectral curves can be seen more visually. Figure 1c,d are the spectral curves after SNV and MSC pretreatment, respectively. The noise interference is significantly reduced in the pretreated spectra.

### 3.2. Division of Calibration Set and Prediction Set

A total of 216 samples were collected, and the data were divided into 2 parts. In order to improve the generalization ability of the model and avoid the bias introduced by manual data segmentation, the current common data division method includes random selection (RS), Kennard–Stone (KS) and sample set partitioning based on joint x-y distances (SPXY) algorithm. Luo et al. [37] reported that the data set divided by the KS algorithm was more prone to overfitting than the SPXY algorithm, and the SPXY algorithm was developed based on the KS algorithm, so this study uses the SPXY algorithm to divide the samples.

### 3.3. Data Distribution and Feature Selection

**Figure 2:**
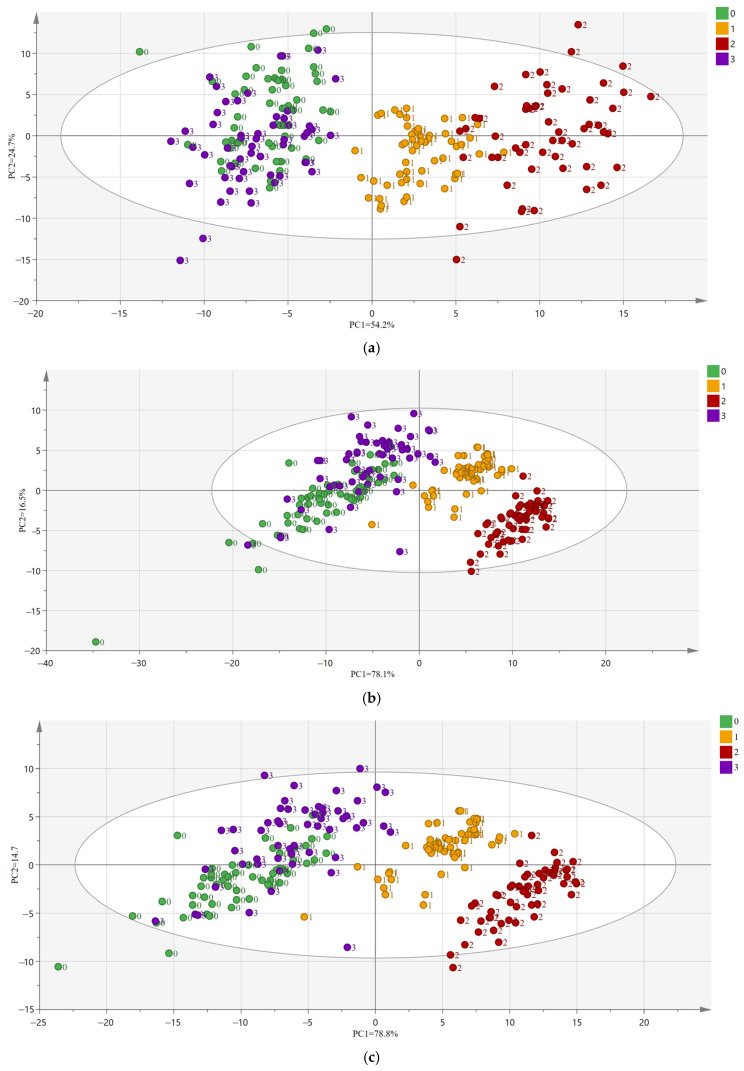
PCA score plots of four oolong teas. (**a**) The PCA of the raw spectra; (**b**) the PCA of the spectra after MSC; and (**c**) the PCA of the spectra after SNV. (0 represents Tieguanyin, 1 represents Maoxie, 2 represents Huangjingui, 3 represents Benshan).

Based on the 104 effective wavelengths of the collected fluorescence hyperspectral information, the two-dimensional characteristics of the four oolong teas are shown in Figure 2: (a) is the PCA of raw spectra, (b) is the PCA of spectra after MSC, and (c) is the PCA of spectra after SNV. In the PCA score plot, each “sample point” represents a sample. The distance between the sample point and origin represents the degree, which is interpreted by PC1 and PC2. The more similar the tea leaves are, the closer the distribution of tea samples will be.

In Figure 2a, the first two principal components (PC1 and PC2) accounted for 78.9% of the total variance, with the highest variations of 54.2% and 24.7%, respectively. In Figure 2b, the first two principal components (PC1 and PC2) accounted for 94.6% of the total variance, with the highest variations of 78.1% and 16.5%, respectively. In Figure 2c, the first two principal components (PC1 and PC2) accounted for 93.5% of the total variance, with the highest variations of 78.8% and 14.7%, respectively. The total contribution of PC1 and PC2 to the variance of MSC and SNV was over 90%, which indicates that the first two PCs are sufficient to explain the total variance of the dataset.

In the PCA score plots of the original spectral data and the preprocessed spectral data, PC1 and PC2 were the first two new variables after the dimensionality reduction from the original spectra, and the score plots were orthogonal and the two variables were independent of each other. Tieguanyin and Benshan are located in the second and third quadrants, while Huangjingui and Maoxie are located in the first and fourth quadrants. The clear separation of Tieguanyin and Benshan was mainly due to the difference in PC2, and the clear separation of Huangjingui and Maoxie was mainly based on the joint action of PC1 and PC2. However, after pretreatment, the separation results of the four teas gradually became obvious, with Maoxie concentrated in the first quadrant, Benshan concentrated in the second quadrant, Tieguanyin concentrated in the third quadrant, and Huangjingui concentrated in the fourth quadrant. Tieguanyin and Benshan were still mixed on the left side of the *y*-axis regardless of whether they were pretreated. These phenomena indicate that PCA can better separate Huangjingui and Maoxie, but Tieguanyin and Benshan are not well distinguished in the PCA.

Noise may be presented in the sample and thus may make subsequent results inaccurate. In the PCA score plot, tolerance ellipses are plotted according to Hotelling. Observations far from the ellipse are outliers. From Figure 2, the outliers were searched, and all samples outside the ellipse were excluded as outliers. According to the markers, among 216 samples, there were 5 outliers in Tieguanyin, 1 in Maoxie (Maoxie was mainly distributed in the first quadrant, and in the preprocessed graph PCA score plot, there was 1 sample in the 3rd quadrant, so that point was excluded), 3 in Benshan and 5 in Huangjingui, leaving 202 samples at last as input for the subsequent model.

**Figure 3:**
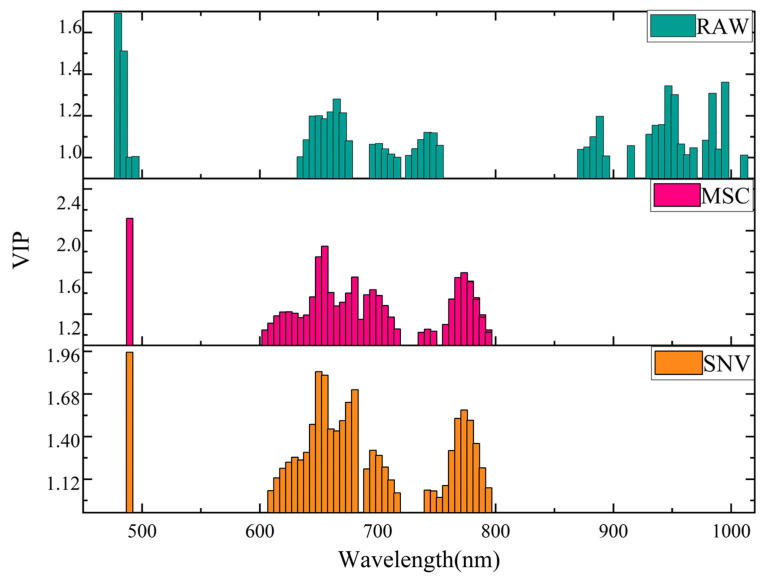
The distributions of all variables in each wavelength after the selection of VIP.

Among the 202 samples screened, there were 104 spectral wavelengths, but not every wavelength played an important role in the subsequent model building. In order to eliminate the interference of irrelevant variables and find out the key wavelengths affecting the classification of oolong tea, the effective wavelengths were selected using the ranking of important variables in PLS–DA, in which the wavelengths with VIP > 1 were taken as the wavelengths carrying important information. Figure 3 shows the distributions of all the variables in each wavelength after the selection of important variables by PLS–DA for the original spectra and the preprocessed spectra. For the un-preprocessed spectral data, 43 features were selected in 104 wavelengths, and 35 and 33 features were selected in MSC and SNV, respectively. These selected wavelengths are relatively evenly distributed, mainly between 600–800 nm, indicating that the key wavelengths affecting oolong tea varieties in the spectral analysis are in this range. These selected wavelengths will be used as input for the subsequent classification models.

### 3.4. Establish Classification Models of Oolong Tea

Table 1 shows the classification results of oolong tea. In all classification results, PLS–DA has higher accuracy than SVM in both calibration and prediction sets. The accuracy of the original spectra and the prediction set of the preprocessed data were 97.22%, 100% and 96.67% in SVM and 98.91%, 100% and 100% in PLS–DA, respectively, while precision and recall were positively proportional to the correspondent accuracy. The prediction of Maoxie was found poor in SVM, and the prediction of Tieguanyin was poorer in PLS–DA, but the prediction improved under both models after preprocessing. Thus, preprocessing is necessary for performing oolong tea classification.

Table 1 also shows the classification results after feature selection (VIP > 1 in PLS–DA). In the SVM, the overall accuracy of the prediction set after direct feature selection of the original spectra was 92.22%, and the overall precision and recall were 92.75% and 92.25%, respectively. The overall accuracy, precision and recall after SNV were 96.67%, 97.00% and 96.75%, respectively; after MSC, all the indexes were 100%. Under the PLS–DA model, the overall accuracy of the prediction set after direct feature selection of the original spectra was 96.67%, and the overall precision and recall were 96.75% and 99.25%, respectively; after SNV and MSC, all the indexes were 100%. These results are superior to the results of the same method in this report [23]. From the above analysis, it can also be seen that PLS–DA is more effective than SVM in classifying oolong tea. The effect of wavelength feature selection after preprocessing is better than that of feature selection directly from the original spectra. Comparing the two preprocessing methods, it can be found that MSC obtained 100% accuracy in both classification models after feature selection. Therefore, MSC_VIP_PLS–DA was selected as the best classification model for this study.

### 3.5. Characteristic Wavelength Analysis

**Figure 4:**
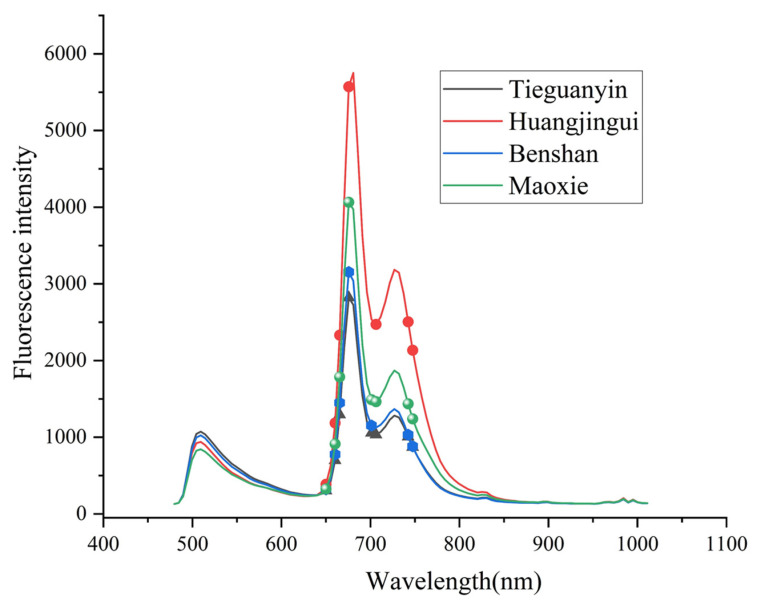
Distribution of key wavelengths in the average spectra.

In order to verify the key wavelengths for tea classification among these features, PLS–DA was used for the key wavelength selection of VIP > 1. Table 2 shows the original spectral data and the preprocessed spectral data after PLS–DA, among which there are 16 identical selected feature wavelengths, namely, 489.54, 634.89, 639.95, 645.03, 650.11, 655.2, 660.29, 665.39, 670.49, 675.6, 701.17, 706.31, 711.44, 716.58, 742.34 and 747.5 nm. These wavelengths were further analyzed for significant differences between different tea samples using ANOVA (*p* < 0.05). Table 3 shows the results of ANOVA. The fluorescence intensities with the same wavelengths of these different teas were significantly different (*p* < 0.05) at 650.11, 660.29, 665.39, 675.6, 701.17, 706.31, 742.34, 747.5 nm. These wavelengths were combined with the mean spectra as shown in Figure 4, from which it can be concluded that the fluorescence intensities of the corresponding wavelengths are consistent with the ANOVA results and the selected wavelengths can represent the differences between oolong tea varieties. According to the analysis of spectral curves in Section 3.1, the differences between these spectra were found to be caused by different internal components, including catechins, theaflavins, anthocyanins, chlorophylls and carotenoids. The key wavelengths here are also mainly concentrated between 650 and 750 nm and thus are consistent with the results of the previous spectral analysis. In [3], the authors used three different wavelength selection methods including BOSS, VISSA and MASS algorithms to screen the wavelengths of different grades of Tieguanyin, and the wavelengths selected by these methods were also concentrated in the range of 600–800 nm, which is consistent with the results obtained in this study and demonstrates the feasibility of using fluorescence hyperspectral techniques in the classification of oolong tea. Therefore, this study can provide key wavelengths for oolong tea classification, and these wavelengths play a key role in the classification model.

## 4. Conclusions

In this study, we not only accurately classified oolong tea but also explored the key wavelengths in the spectra that affect the classification more profoundly. In the process of model building, two preprocessing methods were used to denoise the original spectra, and the spectral data after preprocessing were used to improve the model accuracy in the building of SVM and PLS–DA classification models. To further reduce the influence of redundant wavelengths on the model, PLS–DA (VIP > 1) was used to select the wavelengths, and the selected wavelengths were then used to build the two classification models, and finally, MSC_VIP_PLS–DA was the best model for this classification. To explore the key wavelengths affecting the model, ANOVA was performed on the characteristic wavelengths, and the results showed that the fluorescence intensities were significantly different at 650.11, 660.29, 665.39, 675.6, 701.17, 706.31, 742.34 and 747.5 nm (*p* < 0.05), which corresponded to the spectral curves; it was determined that these wavelengths were the key ones in the classification of oolong tea.

These results suggest that the combination of FHSI and chemometrics is a promising method for the classification of oolong tea, and the exploration of significant differences in wavelengths of oolong tea can identify the key wavelengths affecting tea classification in spectral data at a deeper level. Future work will investigate the relationships between fluorescence spectra in more teas and the internal quality of teas, to bring spectroscopic methods and tea-related research to the forefront.

## Figures and Tables

**Table 1:** Classification results for oolong tea (Tie represents Tieguanyin, Mao represents Maoxie, Huang represents Huangjingui, Ben represents Benshan. Total represents the average accuracy, precision and recall rate of each kind of tea).

Model	Preprocessing	Variables	Class	Calibration Set			Prediction Set		
				Accuracy	Precision	Recall	Accuracy	Precision	Recall
SVM	RAW	104 (none selection)	Tie	95.83%	100.00%	96.00%	100.00%	88.00%	100.00%
			Mao	100.00%	100.00%	100.00%	88.89%	100.00%	89.00%
			Huang	100.00%	100.00%	100.00%	100.00%	100.00%	100.00%
			Ben	100.00%	92.00%	100.00%	100.00%	100.00%	100.00%
			Total	98.96%	98.00%	99.00%	97.22%	97.00%	97.25%
		43 (VIP > 1)	Tie	100.00%	87.00%	100.00%	100.00%	82.00%	100.00%
			Mao	100.00%	100.00%	100.00%	88.89%	89.00%	89.00%
			Huang	100.00%	100.00%	100.00%	100.00%	100.00%	100.00%
			Ben	71.43%	100.00%	71.00%	80.00%	100.00%	80.00%
			Total	92.86%	96.75%	92.75%	92.22%	92.75%	92.25%
	SNV	104 (none selection)	Tie	92.86%	100.00%	93.00%	100.00%	100.00%	100.00%
			Mao	100.00%	100.00%	100.00%	100.00%	100.00%	100.00%
			Huang	100.00%	100.00%	100.00%	100.00%	100.00%	100.00%
			Ben	100.00%	80.00%	100.00%	100.00%	100.00%	100.00%
			Total	98.21%	95.00%	98.25%	100.00%	100.00%	100.00%
		33 (VIP > 1)	Tie	100.00%	100.00%	100.00%	100.00%	88.00%	100.00%
			Mao	100.00%	100.00%	100.00%	100.00%	100.00%	100.00%
			Huang	100.00%	100.00%	100.00%	100.00%	100.00%	100.00%
			Ben	100.00%	100.00%	100.00%	86.67%	100.00%	87.00%
			Total	100.00%	100.00%	100.00%	96.67%	97.00%	96.75%
	MSC	104 (none selection)	Tie	91.67%	100.00%	92.00%	100.00%	88.00%	100.00%
			Mao	100.00%	100.00%	100.00%	100.00%	100.00%	100.00%
			Huang	100.00%	100.00%	100.00%	100.00%	100.00%	100.00%
			Ben	100.00%	83.00%	91.00%	86.67%	100.00%	87.00%
			Total	97.92%	95.75%	95.75%	96.67%	97.00%	96.75%
		35 (VIP > 1)	Tie	100.00%	100.00%	100.00%	100.00%	100.00%	100.00%
			Mao	100.00%	100.00%	100.00%	100.00%	100.00%	100.00%
			Huang	100.00%	100.00%	100.00%	100.00%	100.00%	100.00%
			Ben	100.00%	100.00%	100.00%	100.00%	100.00%	100.00%
			Total	100.00%	100.00%	100.00%	100.00%	100.00%	100.00%
PLS-DA	RAW	104 (none selection)	Tie	100.00%	92.00%	100.00%	95.65%	100.00%	96.00%
			Mao	100.00%	100.00%	100.00%	100.00%	100.00%	100.00%
			Huang	100.00%	100.00%	100.00%	100.00%	100.00%	100.00%
			Ben	84.62%	100.00%	85.00%	100.00%	91.00%	100.00%
			Total	96.15%	98.00%	96.25%	98.91%	97.75%	99.00%
		43 (VIP > 1)	Tie	96.67%	97.00%	97.00%	96.67%	97.00%	97.00%
			Mao	100.00%	100.00%	100.00%	100.00%	100.00%	100.00%
			Huang	100.00%	100.00%	100.00%	100.00%	100.00%	100.00%
			Ben	90.00%	90.00%	90.00%	90.00%	90.00%	100.00%
			Total	96.67%	96.75%	96.75%	96.67%	96.75%	99.25%
	SNV	104 (none selection)	Tie	100.00%	100.00%	100.00%	100.00%	100.00%	100.00%
			Mao	100.00%	100.00%	100.00%	100.00%	100.00%	100.00%
			Huang	100.00%	100.00%	100.00%	100.00%	100.00%	100.00%
			Ben	100.00%	100.00%	100.00%	100.00%	100.00%	100.00%
			Total	100.00%	100.00%	100.00%	100.00%	100.00%	100.00%
		33 (VIP > 1)	Tie	100.00%	100.00%	100.00%	100.00%	100.00%	100.00%
			Mao	100.00%	100.00%	100.00%	100.00%	100.00%	100.00%
			Huang	100.00%	100.00%	100.00%	100.00%	100.00%	100.00%
			Ben	100.00%	100.00%	100.00%	100.00%	100.00%	100.00%
			Total	100.00%	100.00%	100.00%	100.00%	100.00%	100.00%
	MSC	104 (none selection)	Tie	100.00%	100.00%	100.00%	100.00%	100.00%	100.00%
			Mao	100.00%	100.00%	100.00%	100.00%	100.00%	100.00%
			Huang	100.00%	100.00%	100.00%	100.00%	100.00%	100.00%
			Ben	100.00%	100.00%	100.00%	100.00%	100.00%	100.00%
			Total	100.00%	100.00%	100.00%	100.00%	100.00%	100.00%
		35 (VIP > 1)	Tie	100.00%	100.00%	100.00%	100.00%	100.00%	100.00%
			Mao	100.00%	100.00%	100.00%	100.00%	100.00%	100.00%
			Huang	100.00%	100.00%	100.00%	100.00%	100.00%	100.00%
			Ben	100.00%	100.00%	100.00%	100.00%	100.00%	100.00%
			Total	100.00%	100.00%	100.00%	100.00%	100.00%	100.00%

**Table 2:** The wavelengths selected for VIP > 1 in PLS–DA.

Preprocessing Methods	No.	Selected Wavelength
RAW	43	479.65; 484.59; 489.54; 494.49; 634.89; 639.95; 645.03; 650.11; 655.2; 660.29; 665.39; 670.49; 675.6; 696.06; 701.17; 706.31; 711.44; 716.58; 726.86; 732.03; 737.17; 742.34; 747.5; 752.65; 872.69; 877.95; 883.22; 888.51; 893.79; 914.95; 930.88; 936.2; 941.5; 946.84; 952.16; 957.5; 962.84; 968.16; 978.86; 984.23; 989.57; 994.94; 1011.05
MSC	35	489.54; 604.51; 609.56; 614.61; 619.69; 624.75; 629.81; 634.89; 639.95; 645.03; 650.11; 655.2; 660.29; 665.39; 670.49; 675.6; 680.7; 685.83; 690.94; 696.06; 701.17; 706.31; 711.44; 716.58; 737.17; 742.34; 747.5; 757.85; 763; 768.2; 773.39; 778.55; 783.75; 788.95; 794.15
SNV	33	489.54; 609.56; 614.61; 619.69; 624.75; 629.81; 634.89; 639.95; 645.03; 650.11; 655.2; 660.29; 665.39; 670.49; 675.6; 680.7; 690.94; 696.06; 701.17; 706.31; 711.44; 716.58; 742.34; 747.5; 752.65; 757.85; 763; 768.2; 773.39; 778.55; 783.75; 788.95; 794.15;

**Table 3:** ANOVA results for each wavelength.

Wavelength/nm	489.54	634.89	639.95	645.03	650.11	655.2	660.29	665.39
Tieguanyin	231.56 ± 24.01 ^a^	231.77 ± 16.31 ^a^	231.85 ± 16.18 ^a^	254.21 ± 16.59 ^a^	317.14 ± 17.33 ^a^	484.37 ± 17.93 ^a^	897.98 ± 20.66 ^a^	1772.93 ± 25.47 ^a^
Maoxie	240.07 ± 14.20 ^a^	249.03 ± 10.93 ^a^	253.06 ± 11.19 ^b^	276.64 ± 11.26 ^b^	339.49 ± 10.54 ^b^	507.87 ± 12.00 ^a^	923.06 ± 15.42 ^b^	1793.24 ± 26.15 ^b^
Huangjingui	234.42 ± 13.73 ^a^	233.23 ± 10.53 ^b^	240.62 ± 10.61 ^c^	266.39 ± 11.15 ^c^	330.84 ± 11.34 ^c^	484.58 ± 12.33 ^b^	845.42 ± 15.34 ^c^	1584.60 ± 22.11 ^c^
Benshan	253.42 ± 29.82 ^b^	246.92 ± 21.95 ^b^	250.95 ± 21.97 ^c^	278.50 ± 21.93 ^c^	350.06 ± 23.24 ^d^	528.76 ± 24.87 ^c^	954.03 ± 29.64 ^d^	1845.04 ± 51.84 ^d^
**Wavelength/nm**	**670.49**	**675.6**	**701.17**	**706.31**	**711.44**	**716.58**	**742.34**	**747.5**
Tieguanyin	3058.43 ± 35.10 ^a^	4015.49 ± 37.25 ^a^	1421.99 ± 30.72 ^a^	1389.86 ± 31.68^a^	1441.51 ± 34.26 ^a^	1541.62 ± 36.67 ^a^	1348.80 ± 44.45 ^a^	1150.73 ± 41.73 ^a^
Maoxie	3074.16 ± 40.44 ^b^	4062.58 ± 44.93 ^b^	1496.39 ± 33.07 ^b^	1471.07 ± 33.73 ^b^	1534.77 ± 34.12 ^a^	1650.03 ± 34.87 ^a^	1443.74 ± 33.87 ^b^	1247.50 ± 30.70 ^b^
Huangjingui	2708.93 ± 35.92 ^b^	3672.91 ± 43.78 ^c^	1715.80 ± 29.71 ^c^	1674.04 ± 29.10 ^c^	1734.17 ± 29.06 ^b^	1859.20 ± 30.27 ^b^	1691.45 ± 45.21 ^c^	1453.68 ± 40.66 ^c^
Benshan	3142.46 ± 87.62 ^c^	4103.93 ± 99.52 ^d^	1446.08 ± 57.81 ^d^	1410.60 ± 59.42 ^d^	1455.16 ± 64.79 ^c^	1546.21 ± 71.97 ^c^	1282.70 ± 66.33 ^d^	1086.56 ± 57.35 ^d^

Data represent the mean ± standard deviation. Statistical analysis was carried out by analysis of variance and post-Duncan test, and different lowercase letters (a–d) were used to indicate the importance of statistical signals (*p* < 0.05). The same letter means there is no significant difference between the teas, and different letters mean there is a significant difference.

## Data Availability

This data can be found here: https://github.com/guyueguyue/guyuea/compare/main...guyueguyue-patch-1?quick_pull=1#diff-d28270bf1337e1ec31a4e067e21dfb340633288db0270a97d60288145908727b (accessed on 4 August 2022).

## Abbreviations

The following abbreviations are used in this manuscript:

FHSI	Fluorescence hyperspectral technology
SNV	Standard normal variation
MSC	Multiple scatter correction
PCA	Principal component analysis
VIP	Variable importance for the projection
PLS-DA	Partial least squares discriminant analysis
SVM	Support vector machine
GC-MS	Gas chromatography-mass spectrometry
ICP-MS	Inductively coupled plasma mass spectrometry
ASAP-MS	Atmospheric solids analysis probe-mass spectrometry
KNN	K-nearest neighbor
HCA	Hierarchical cluster analysis
LDA	Linear discriminant analysis
SIMCA	Soft independent modelling of class analogies
Vis-NIR	visible near-infrared
RS	Random selection
KS	Kennard–Stone
SPXY	Sample set partitioning based on joint x-y distances
ANOVA	Analysis of variance
